# Value of Anterior Band of the Inferior Glenohumeral Ligament Area as a Morphological Parameter of Adhesive Capsulitis

**DOI:** 10.1155/2019/9301970

**Published:** 2019-05-07

**Authors:** Yun-Sic Bang, Junbeom Park, Sun Young Lee, Jiyeon Park, Sukhee Park, Young Joo, Young Uk Kim, Yoon Kyung Lee

**Affiliations:** ^1^Department of Anesthesiology and Pain Medicine, CHA Bundang Medical Center, CHA University, Seongnam, Republic of Korea; ^2^Department of Anesthesiology and Pain Medicine, Catholic Kwandong University of Korea, College of Medicine, International ST. Mary's Hospital, Incheon, Republic of Korea; ^3^Department of Anesthesiology and Pain Medicine, Kangdong Sacred Heart Hospital, Hallym University College of Medicine, Seoul, Republic of Korea

## Abstract

**Objective:**

Thickened inferior glenohumeral ligament (IGHL) is considered as one of the major morphological parameters of adhesive capsulitis (AC). Previous studies reported that the anterior band of inferior glenohumeral ligament thickness (aIGHLT) is correlated with shoulder capsular contracture, luxatio erecta humeri, and AC. However, the thickness varies from the measured angle. To reduce this measurement error, we devised a new morphological parameter, called the anterior band of inferior glenohumeral ligament area (aIGHLA).

**Methods:**

The aIGHL samples were collected from 54 patients with AC and from 50 control subjects who underwent shoulder magnetic resonance imaging (MRI) without any evidence of AC. Coronal T2-weighted MRI images were obtained at the shoulder level from each patient. We measured the aIGHLA and aIGHLT at the maximal view of the IGHL in the coronal plane using our picture archiving and communication system. The aIGHLA was measured at the whole cross-sectional area of the IGHL in the most hypertrophied segment of the coronal MR images. The aIGHLT was measured at the thickest point of the IGHL.

**Results:**

The average aIGHLA was 55.58 ± 14.16 mm^2^ in the control group and 83.71 ± 28.45 mm^2^ in the AC group. The average aIGHLT was 3.47 ± 0.99 mm in the control group and 4.52 ± 1.02 mm in the AC group. AC patients showed significantly greater aIGHLA (*p* < 0.001) and aIGHLT (*p* < 0.001) than control subjects. Receiver operating characteristic (ROC) curve analysis showed that the optimal cut-off score of the aIGHLA was 63.37 mm^2^, with 79.6% sensitivity, 80.0% specificity, and AUC of 0.84 (95% CI, 0.76–0.92). The optimal cut-off point of the IGHLT was 3.81 mm, with 74.1% sensitivity, 74.0% specificity, and AUC of 0.77 (95% CI, 0.68–0.86).

**Conclusions:**

Although the aIGHLA and aIGHLT were both significantly associated with AC, the aIGHLA was a more sensitive diagnostic parameter.

## 1. Introduction

Adhesive capsulitis (AC) of shoulder describes a pathological process, in which the body forms adhesions or excessive scar tissue across the glenohumeral (GH) joint, leading to stiffness, dysfunction, and pain. It is a debilitating condition that occurs spontaneously (idiopathic or primary AC) or following shoulder trauma during surgery (secondary AC) [1]. Although most AC patients are successfully treated with nonoperative care, years can ensue (mean, 1.5–3 years) before recovering normal range of motion (ROM) [2, 3]. Prompt diagnosis of AC and effective treatment with intra-articular corticosteroid injection or physical therapy shortens the duration of joint pain and stiffness and significantly reduces patient morbidity [2, 4]. The diagnosis of AC still remains clinical [5, 6]. The diagnostic criteria for AC include shoulder stiffness lasting more than one month, shoulder pain, and absence of other abnormalities [2, 3]. In the presence of established physical signs and typical symptoms, the clinical evaluation of AC has high diagnostic sensitivity and specificity [1, 7, 8]. However, the early phases of AC are characterized by variable symptoms and diagnostic challenges [3]. Imaging modalities play an important role in the diagnosis of AC in the presence of atypical clinical symptoms. The inferior glenohumeral ligament (IGHL) complex comprises three components supporting the inferior aspect of the shoulder. It consists of an anterior band, a posterior band, and an interposed axillary pouch [9]. The IGHL complex stabilizes the shoulder, and its function depends on the position of the shoulder. With the arm in a 90° abduction and external rotation, the anterior band of the IGHL is the main glenohumeral joint stabilizer [9, 10]. An abnormal anterior band of IGHL (aIGHL) is a major finding of AC [11]. Magnetic resonance imaging (MRI) facilitates the pathological evaluation of the aIGHL and other associated pathological findings associated with the shoulder joint [12]. Previous studies investigated the aIGHL based on a single measurement at the approximate “middle” or “halfway” of the aIGHL [11]. However, partial tear and asymmetrical thickening of the aIGHL may be detected widely, suggesting the scope for measurement error(s). In contrast to aIGHLT, the anterior band of inferior glenohumeral cross-sectional area (aIGHLA) measures the whole cross-sectional area of the aIGHL without the possibility of measurement error. Thus, for evaluation of hypertrophy of the whole IGHL, we developed a new morphological parameter, called the aIGHLA. We hypothesized that the aIGHLA is a key morphological parameter in AC diagnosis. Therefore, we compared the aIGHLA and aIGHLT between AC patients and normal controls using shoulder MRI.

## 2. Methods

### 2.1. Patients

This study was registered at the University of Catholic Kwandong, Republic of Korea, Incheon (IS18RISI0014). The Institutional Review Board (IRB) reviewed and approved the research protocol. We retrospectively reviewed patients who visited our pain clinic from November 2015 to November 2017 and who were diagnosed with AC.

The inclusion criteria were as follows: (1) ache or dull pain in the outer shoulder area; (2) available shoulder MRI image(s); (3) severe restriction in shoulder movement; (4) progressive loss of both passive and active range of motion (ROM); (5) symptoms present for at least 3 months; and (6) medical charts containing follow-up data of AC patients were analyzed for confirmation. We excluded patients if they had any of the following conditions: (1) history of shoulder surgery; (2) history of rheumatoid arthritis; (3) calcific tendinitis; (4) full-thickness rotator cuff tear; and (4) any neuromuscular disease.

A total of 54 patients who met the inclusion criteria were enrolled after an experienced board-certified musculoskeletal radiologist confirmed the diagnosis of AC.

There were 20 (37.0%) men and 34 (63.0%) women with a mean age of 56.98 ± 7.16 years (range, 41 to 74 years) (Table 1). All patients underwent shoulder MRI. To compare the aIGHLA and aIGHLT between patients with and without AC, we also enrolled a control group of subjects who underwent shoulder MRI, without any evidence of AC from November 2015 to November 2017. We only enrolled patients in the control group who did not have AC-related symptoms. The control group included 50 individuals (19 men and 31 women) with a mean age of 56.44 ± 5.52 years (range, 44 to 65 years).

### 2.2. Imaging Parameters

MRI analyses were performed with 3.0T Magnetom Skyra MRI system (Siemens Healthcare, Erlangen, Germany) and 3T Ingina (Philips, Eindhoven, Netherlands) scanners. For all MRI examinations, we obtained sagittal and coronal T2-weighted images with <3 mm slice thickness, 0.9-mm intersection gap, 4010-ms/76-ms repetition time (TR)/echo time (TE), 150 × 150 field of view, 512 × 256 matrix, and >3 echo train length (ETL).

### 2.3. Image Analysis

**Figure 1:**
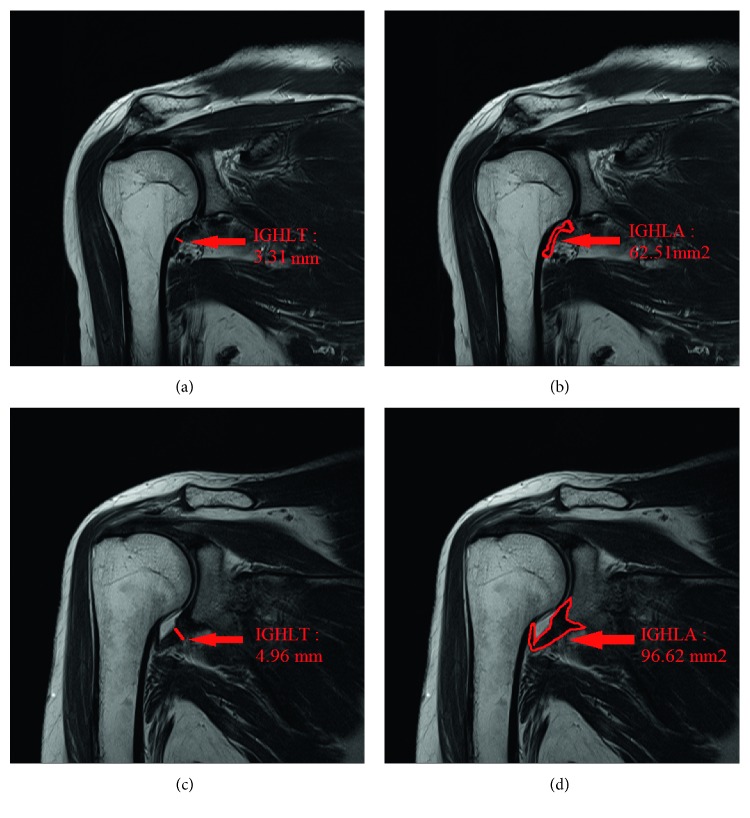
T2-weighted turbo-spin-echo coronal MR image in inferior glenohumeral ligament sections: (a) normal aIGHLT; (b) normal aIGHLA; (c) aIGHLT in the AC group; (d) aIGHLA in the AC group. aIGHLT = anterior band of inferior glenohumeral ligament thickness. aIGHLA = anterior band of inferior glenohumeral ligament area. AC = adhesive capsulitis.

The aIGHLT and aIGHLA measurements were performed by the same physician, who was blinded to the diagnosis of the AC. T2-weighted turbo-spin-echo coronal MR images were obtained at the thickest visualization of aIGHL. *We measured the aIGHLA and aIGHLT on MRI using a picture archiving and communication system (INFINITT; Infinitt Healthcare, Seoul, Korea). INFINITT system offers an enterprise imaging solution with exact diagnostic viewer. The aIGHLA was measured as the whole cross-sectional area of the IGHL at the thickest point.* The aIGHLT was measured at the thickest point between the origin and insertion (Figures 1(a)–1(d)).

### 2.4. Statistical Analysis

Data were presented as mean ± standard deviation (SD). We compared the aIGHLA and aIGHLT between the AC and control groups using unpaired *t*-tests. The validity of the aIGHLA and aIGHLT for diagnosis of AC was estimated by receiver operator characteristic (ROC) curves, area under the curve (AUC), cut-off values, sensitivity, and specificity with 95% confidence intervals (CIs). *P* values less than 0.05 were considered statistically significantly different. SPSS for Windows version 22 (IBM SPSS Inc., Chicago, IL) was used for the statistical analysis.

## 3. Results

**Figure 2:**
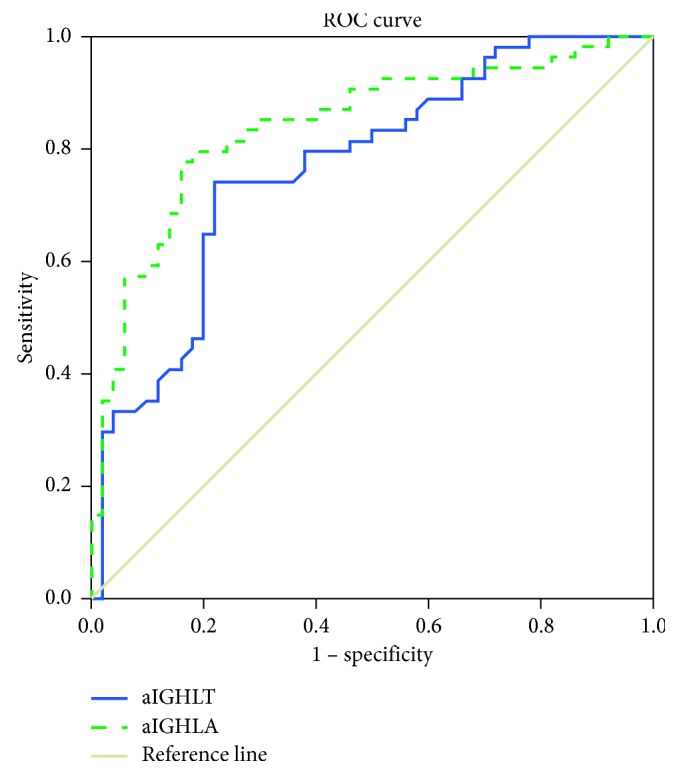
Receiver operating characteristic (ROC) curve of anterior band of inferior glenohumeral ligament thickness (aIGHLT) and anterior band of inferior glenohumeral ligament area (aIGHLA) for prediction of adhesive capsulitis. The best cut off point of aIGHLA was 63.37 mm^2^ versus 3.81 mm of aIGHLT, with sensitivity 79.6% versus 74.1%, specificity 80.0% versus 74.0% and AUC 0.84 versus 0.77, respectively. aIGHLT AUC (95% CI) = 0.77 (0.68–0.86). aIGHLA AUC (95% CI) = 0.84 (0.76–0.92). AUC = area under the curve.

AC affected 29 right shoulders and 25 left shoulders of patients. The average aIGHLA was 55.58 ± 14.16 mm^2^ in the control group and 83.71 ± 28.45 mm^2^ in the AC group. The average aIGHLT was 3.47 ± 0.99 mm in the control group and 4.52 ± 1.02 mm in the AC group. AC patients had significantly greater aIGHLA (*p* < 0.001) and aIGHLT (*p* < 0.001) than control subjects (Table 1). The ROC curve analysis (Figure 2) showed that the optimal cut-off point of the IGHLT was 3.81 mm, with 74.1% sensitivity, 74.0% specificity, and AUC of 0.77 (95% CI, 0.68–0.86) (Table 2). The optimal cut-off score of the aIGHLA was 63.37 mm^2^, with 79.6% sensitivity, 80.0% specificity, and AUC of 0.84 (95% CI, 0.76–0.92) (Table 3).

## 4. Discussion

AC is a common clinical condition characterized by global limitation of passive and active ROM and pain in the affected shoulder [7, 13]. AC was challenging in previous histological studies without evidence of significant inflammatory focus [14]. Several types of conservative management at primary care facilities have not proven effective in most AC patients [14]. The primary objective of the management of AC associated with stiffness is to restore or improve the shoulder ROM. Various imaging modalities, such as arthrogram, ultrasonography, and Doppler ultrasound, facilitate precise diagnosis [12, 15]. MRI is also commonly used to investigate shoulder disorders and exclude concomitant conditions [13]. It has been used to identify morphological changes in the joint capsule at the axillary pouch level with variable results and the joint capsule and periarticular tissues (obliteration of adjacent fat planes, contrast enhancement, and thickening) at the rotator interval [16, 17].

However, diagnosis is still a challenge, especially due to the lack of reliable diagnostic standards for AC. Based on clinical and experimental investigations, the contracture of the aIGHL restricting flexion and internal rotation was confirmed [18]. The aIGHL is the main glenohumeral joint stabilizer [9]. Thus, aIGHL injury is associated with clinically evident instability [19]. The aIGHL arises from the anterior inferior labrum. It is important clinically for AC diagnosis [20]. The normal aIGHL appears as low-signal intensity bands, stretching from the inferior labrum to the humeral neck on MR coronal view [9, 19]. The anterior band is best seen on anterior coronal image through the glenohumeral joint. Gondim Teixeira et al. demonstrated that changes in signal intensity at the aIGHL are the key parameters in the diagnosis of AC on conventional MRI [3]. Passanante et al. insisted that aIGHL injuries are the most important cause of shoulder pain [9]. Michelin et al. reported that the aIGHL is thickened in AC patients, and ultrasound enables the measurement of aIGHL thickness in the axilla [11]. The average thickness of aIGHL using ultrasound was 4.0 mm in an AC shoulder compared with 1.3 mm in the asymptomatic contralateral shoulder. However, these studies did not compare normal subjects and AC. Our results demonstrated that the aIGHLT of normal subjects was 3.47 ± 0.99 mm and in patients with AC, it was 4.52 ± 1.02 mm.

As discussed above, previous studies focused only on the aIGHLT [11]. However, the wide range of locations of partial tear and asymmetrical thickening of the aIGHL leads to measurement errors. By contrast, the aIGHLA is not associated with such error as it measures the whole cross-sectional area of the IGHL.

We hypothesized that the coronal cross-sectional area of the aIGHLA may predict AC. Due to the absence of measurement errors, the aIGHLA is better than the aIGHLT as a morphological parameter of AC. In the current study, we found that the aIGHLA had 79.6% sensitivity, 80.0% specificity, and AUC of 0.84 (95% CI, 0.76–0.92) to predict AC. By contrast, the aIGHLT had 74.1% sensitivity, 74.0% specificity, and AUC of 0.77 (95% CI of 0.68–0.86). These findings suggest that the aIGHLA is a better predictor of AC than the aIGHLT.

Our present study has several limitations. The IGHL consists of an anterior band, a posterior band, and an interposed axillary pouch. However, we focused only on aIGHL because of its prime importance in the stability of the shoulder joint [12].

Second, there are several morphological abnormalities of AC, such as coracohumeral ligament contracture, changes in the appearance of the shoulder joint capsule, or thickened periarticular tissues, which are effective in discriminating AC [3, 14, 21–25]. However, we only assessed the measurement of aIGHLA and aIGHLT on MRI. Third, errors may be associated with the shoulder MRI measurement of aIGHLA and aIGHLT. Although we measured these morphological parameters in the coronal plane that best showed the ATFL, the coronal images used for the analysis of the variables may be inhomogeneous because of differences in the cutting angle or MRI level due to technical reasons and individual anatomic variation. In addition, a 3 mm slice of axial T2-weighted TRE MR image is thicker than the ideal slice. Thus, small lesions are frequently difficult to detect. Fourth, the principal methodological limitation was the retrospective evaluation of the medical records for data analysis [26–28]. Despite these weaknesses, this is the first study to document that the aIGHLA is associated with AC.

## 5. Conclusion

Although aIGHLA and aIGHLT are both significantly associated with AC, the aIGHLA is a more sensitive diagnostic parameter for AC than aIGHLT. We identified the optimal cut-off point of the aIGHLA as 63.37 mm^2^, with 79.6% sensitivity and 80.0% specificity. The best cut-off point of the aIGHLT was 3.81 mm, with 74.1% sensitivity and 74.0% specificity. When evaluating patients with AC, physicians should carefully assess the aIGHLA rather than the aIGHLT.

## Figures and Tables

**Table 1:** Comparison of the characteristics of control and AC groups.

Variable	Control group *n*=50	AC group *n*=54	Statistical significance
Gender (male/female)	19/31	20/34	NS
Shoulder image (Rt/Lt)	28/22	29/25	NS
Age (yrs)	56.44 ± 5.52	56.98 ± 7.16	NS
aIGHLT (mm)	3.47 ± 0.99	4.52 ± 1.02	*p* < 0.001
aIGHLA (mm^2^)	55.58 ± 14.16	83.71 ± 28.45	*p* < 0.001

Data represent the mean ± standard deviation (SD) or the numbers of patients. Abbreviations: AC, adhesive capsulitis; aIGHLT, anterior band of inferior glenohumeral ligament thickness; aIGHLA, anterior band of inferior glenohumeral ligament area; NS, not statistically significant (*p* > 0.05).

**Table 2:** Sensitivity and specificity of each cut-off point of the aIGHLT.

aIGHLT (mm)	Sensitivity (%)	Specificity (%)
2.19	100	8
2.58	98.1	22.0
3.07	88.9	34.0
3.81^a^	74.1	74.0
4.48	48.1	80.0
7.94	0	100

^a^The best cut-off point on the receiver operating characteristic (ROC) curve. Abbreviations: aIGHLT, anterior band of inferior glenohumeral ligament thickness.

**Table 3:** Sensitivity and specificity of each cut-off point of the aIGHLA.

aIGHLA (mm^2^)	Sensitivity (%)	Specificity (%)
35.26	100	8
49.03	94.4	32.0
54.05	90.7	50
63.37^a^	79.6	80.0
67.06	68.5	86.0
113.27	14.8	100

^a^The best cut-off point on the receiver operating characteristic (ROC) curve. Abbreviations: aIGHLA, anterior band of inferior glenohumeral ligament area.

## Data Availability

The data used to support the findings of this study are available from the corresponding author upon request.
